# Characteristics of Small Bowel Polyps Detected in Cowden Syndrome by Capsule Endoscopy

**DOI:** 10.1155/2015/475705

**Published:** 2015-06-24

**Authors:** Keita Saito, Eiki Nomura, Yu Sasaki, Yasuhiko Abe, Nana Kanno, Naoko Mizumoto, Rika Shibuya, Kazuhiro Sakuta, Makoto Yagi, Kazuya Yoshizawa, Daisuke Iwano, Takeshi Sato, Shoichi Nishise, Yoshiyuki Ueno

**Affiliations:** Department of Gastroenterology, Faculty of Medicine, Yamagata University, 2-2-2 Iida-Nishi, Yamagata 990-9585, Japan

## Abstract

Cowden syndrome is an uncommon, autosomal dominant disease characterized by multiple hamartomas and hyperplastic lesions in the skin, mucous membrane, brain, breast, thyroid, and gastrointestinal tract. About 30% of Cowden syndrome cases are reportedly complicated by malignant diseases. Hamartomatous polyps occur throughout the gastrointestinal tract, the most common sites being the stomach, colon, esophagus, and duodenum. Small bowel polyps can occur in Cowden syndrome; however, they are difficult to detect by conventional examination, including double-contrast X-ray study. Here, we report three cases of Cowden syndrome with small bowel polyps, which were detected by capsule endoscopy. The small bowel polyps of Cowden syndrome frequently occur at the oral end of the small bowel, especially in the duodenum and jejunum, and their color is similar to that of the surrounding mucosa; additionally, the polyps are relatively small (2–5 mm). Capsule endoscopy is useful for detecting small bowel polyps in Cowden syndrome.

## 1. Introduction

Cowden syndrome is an uncommon, autosomal dominant disease characterized by multiple hamartomas and hyperplastic lesions of the skin, mucous membrane, brain, breast, thyroid, and gastrointestinal tract [1, 2]. Its incidence is estimated to be one in 200,000–250,000 [3]. About 30% of Cowden syndrome cases are reportedly complicated by malignant tumors [4].

The incidence of gastrointestinal polyps is 65.6% in the esophagus, 75% in the stomach, 36.5% in the duodenum, and 65.6% in the colon [5]. The small bowel polyps can occur in Cowden syndrome; however, the characteristics of these polyps are unclear, and they are difficult to detect by conventional examination, including double-contrast X-ray study [6].

We report three cases of Cowden syndrome with small bowel polyps, which were detected by capsule endoscopy (CE), and describe the characteristic findings of the small bowel polyps in this syndrome.

## 2. Case Reports

### 2.1. Case  1

**Figure 1:**
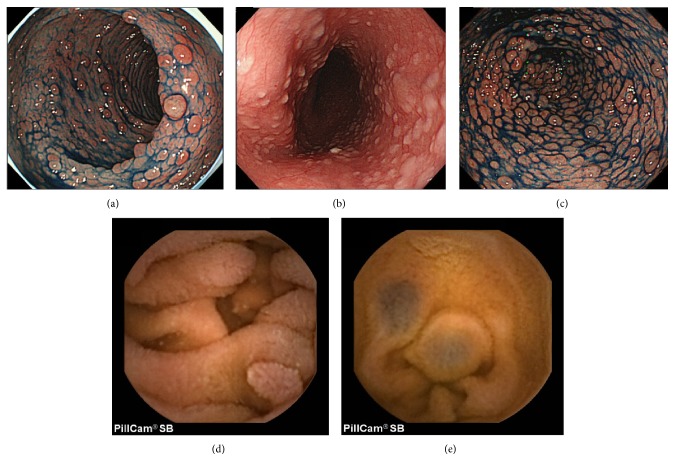
Endoscopic views of Case 1. (a) Colonoscopy revealed multiple rectal polyps. (b) Esophagogastroduodenoscopy (EGD) showed whitish polypoid lesions in the esophagus. (c) EGD showed multiple gastric polyps. (d) Capsule endoscopy revealed multiple polypoid lesions similar in color to the surrounding mucosa in the jejunum, with their diameters of 2–5 mm. (e) Capsule endoscopy revealed hemangiomas in the jejunum.

A 46-year-old man was referred to our hospital for hematochezia. He had no significant medical history and family history. He had multiple facial papules and small, whitish gingival papilloma. A colonoscopy revealed multiple rectosigmoid colon polyps, predominantly located in the lower rectum (Figure 1(a)). Esophagogastroduodenoscopy (EGD) showed whitish polypoid lesions in the esophagus (Figure 1(b)) and multiple gastric polyps (Figure 1(c)). Biopsy specimens from the gastric and rectal polyps revealed hamartomatous changes and hyperplasia. The esophageal polyps were diagnosed histopathologically as glycogenic acanthosis. The facial papules were diagnosed as trichilemmomas by histopathological examination. He was diagnosed with Cowden syndrome in accordance with the criteria of the International Cowden Consortium [7]. CE was performed to examine the small bowel and revealed multiple polypoid lesions that were similar in color to the surrounding mucosa; their diameters ranged from 2 to 5 mm at the distal end of the duodenum and jejunum (Figure 1(d)). The polyps were sparse, although their numbers were higher in the jejunum. Several hemangiomas were also observed in the jejunum (Figure 1(e)). They were more frequently observed at the oral end of the small bowel. The duodenal polyps were histopathologically diagnosed as being hamartomatous. No further malignant complications were observed; the patient was followed up in our hospital.

### 2.2. Case  2

**Figure 2:**
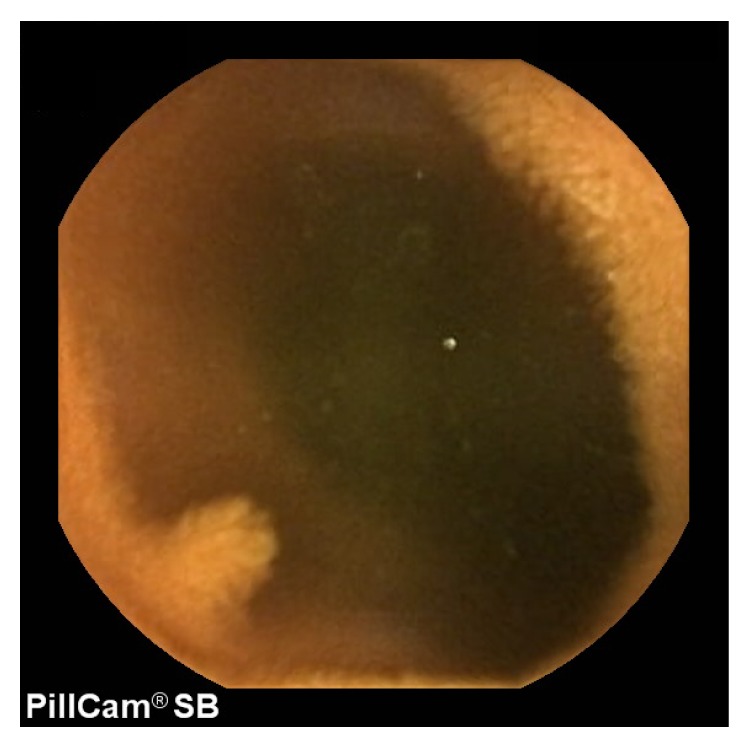
Capsule endoscopy revealed polyps of normal color in the jejunum.

A 60-year-old woman was referred to our hospital for further examination of multiple gastric polyps. She had a past history of breast fibroadenoma and thyroid goiter. She had oral papilloma, esophageal glycogenic acanthosis, and polyposis in the stomach, duodenum, and colon as observed by endoscopic examination. Histological assessment of the biopsy specimens revealed that the gastric and colonic polyps were hamartomatous, and she was diagnosed with Cowden syndrome. CE revealed many polyps of normal color that ranged from 2 to 5 mm in size in the small bowel (Figure 2). These polyps were sparse but were more frequently observed in the jejunum.

### 2.3. Case  3 (Daughter of Case  2)

**Figure 3:**
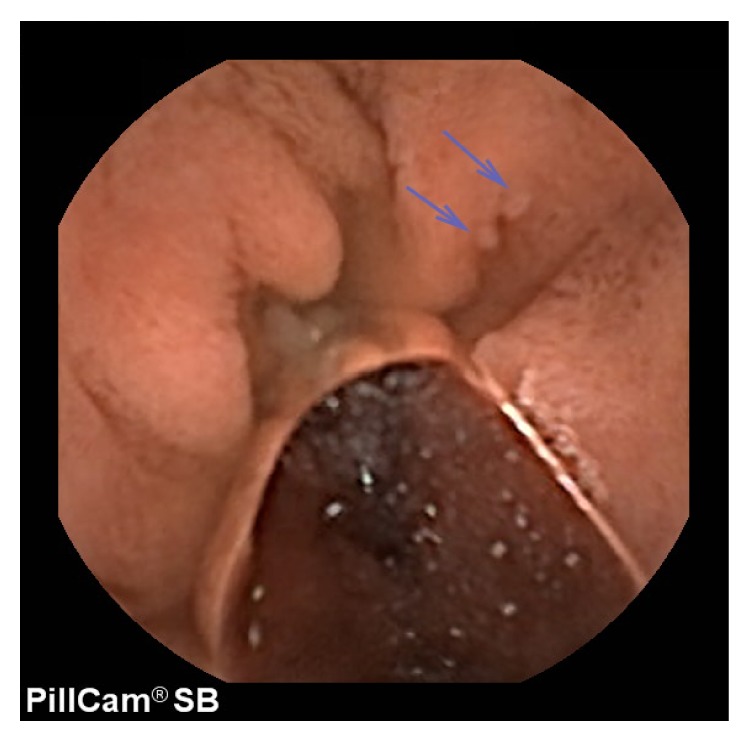
Capsule endoscopy revealed minimal polyps of normal color in the duodenum.

A 27-year-old woman received gastrointestinal examination after her mother's diagnosis with Cowden syndrome. EGD revealed esophageal multiple glycogenic acanthosis and duodenal polyps, but no significant lesions were found in the stomach, unlike her mother. A colonoscopy revealed small hamartomatous polyps in the rectum. She had bilateral tonsil papilloma, multiple thyroid cysts, and breast lipoma. She was diagnosed with Cowden syndrome. CE revealed minimal polyps of normal color, which ranged from 2 to 3 mm in size, from the duodenum to the oral end of the jejunum (Figure 3). We did not find any significant lesions in the ileum or malignant tumors in her body.

## 3. Discussion

Cowden syndrome, also known as multiple hamartoma syndrome, was first described in 1963 by Lloyd and Dennis [1]. This uncommon syndrome is characterized by multiple hamartomas and hyperplastic lesions of the whole body [2]. About 30% of Cowden syndrome cases are reportedly complicated by malignant diseases, including breast cancer, thyroid cancer, endometrium cancer, renal cell cancer, colorectal cancer, and melanoma [2–4].

Cowden syndrome is an autosomal dominant disorder that has been linked to germline mutations in the* PTEN* (phosphatase and tensin homolog) gene located on chromosome 10q23.3 [3]. Approximately 80% of patients with classically defined Cowden syndrome carry the* PTEN* gene [8], which acts as a negative regulator of the PI3-kinase signaling pathway by catalyzing the dephosphorylation of PIP3 [9].* PTEN* hamartoma tumor syndrome incorporates several rare diseases that develop secondary to germline mutations within the* PTEN* gene. Component syndromes include Cowden syndrome and Bannayan-Riley-Ruvalcaba syndrome, which many now consider to be a single entity with age-related phenotypic presentations [10]. In our patients, genetic analysis was not performed.

The diagnosis of Cowden syndrome was originally made based on skin examination and family history [11]. However, the original diagnostic criteria of the International Cowden Consortium are now commonly used [7]. The presence of gastrointestinal polyposis is considered as a minor criterion owing to the lack of systematic studies to determine its true frequency and histology [12]. Nonetheless, in reality, it is a very common finding, with an estimated prevalence of up to 80% in patients with Cowden syndrome. In particular, esophageal polyps composed of glycogenic acanthosis are reportedly characteristic of Cowden syndrome [13, 14]. All three cases reported here fulfilled the criteria of the International Cowden Consortium. Gastric polyposis was found in Cases 1 and 2 and rectal polyposis and esophageal polyposis were found in all three. Esophageal polyposis was histopathologically shown to be composed of glycogenic acanthosis.

Small bowel polyps can arise in Cowden syndrome. However, the characteristics of these polyps are unclear, and they are difficult to detect with conventional examination, including double-contrast X-ray study, due to the small size of the polyps and the fact that they do not protrude much [6]. These polyps have been histopathologically found to be hamartomatous or hyperplastic polyps [2]. CE allows for endoscopic imaging of the entire small bowel without discomfort [15]. Three previous case reports have demonstrated small bowel polyps in Cowden syndrome using CE [6, 16, 17]. Nakaji et al. performed CE on a 24-year-old man with Cowden syndrome and observed multiple polypoid lesions that ranged from 3 to 5 mm in size in the small bowel, with the number of these polyps increasing from the jejunum to the terminal ileum [6]. Further, Riegler et al. reported a 53-year-old female with four minimal polyps in different tracts of the jejunum and vascular ectasia in the ileum as detected by CE [16]. Additionally, Hatogai et al. reported that small bowel polyps in Cowden syndrome are more clearly visualized using contrast image CE [17]. To our knowledge, there have been no previous case series of small bowel polyps in Cowden syndrome as demonstrated by CE. In our series, small bowel polyps were detected in all three cases. In Case 1, multiple polypoid lesions were found of a similar color to the surrounding mucosa, with their diameters ranging from 2 to 5 mm in the duodenum and jejunum. The polyps were sparse, although their numbers were higher in the jejunum. Several hemangiomas were also observed in the jejunum. Hemangiomas were frequently observed at the oral end of the small intestine. Many polyps of normal color, ranging from 2 to 5 mm in size, were observed in the small bowel in Case 2, mostly in the jejunum. Minimal polyps were seen in the duodenum to the jejunum in Case 3. Histopathological examination revealed hamartomatous polyps in all three cases, which needed to be biopsied.

In all the three cases, preparation for CE consisted solely of fasting (no solid food, only clear liquids) for 12 h prior to the procedure, and polyethylene glycol solution was not used; nonetheless, we obtained relatively clear images from the jejunum to the terminal ileum. It was reported that ileal involvement is not rare [12] and that polyp density increased aborally [6]; however, Riegler et al. [16] showed jejunal polyps, not ileal polyps, in Cowden syndrome. The quality of bowel preparation and imaging could affect polyp detection by CE; nevertheless, we think that there were more jejunal polyps than ileal polyps in our patients. Further examinations are needed to clarify the most common sites for small bowel polyps in Cowden syndrome.

We did not detect any malignant diseases in the three cases. However, Cowden syndrome is associated with increased susceptibility to malignant diseases, and periodic follow-up examination and early diagnosis are necessary.

In summary, we described the characteristics of small bowel polyps in Cowden syndrome using CE. Small bowel polyps in Cowden syndrome are frequently observed at the oral end of the small bowel, especially in the duodenum and jejunum, and their color is similar to that of the surrounding mucosa; additionally, the polyps are relatively small (2–5 mm). CE is useful for detecting polyps in the small bowel in Cowden syndrome.
